# Kirigami‐Inspired Deformable 3D Structures Conformable to Curved Biological Surface

**DOI:** 10.1002/advs.201801070

**Published:** 2018-10-16

**Authors:** Chao Yang, Heng Zhang, Youdi Liu, Zhongliang Yu, Xiaoding Wei, Youfan Hu

**Affiliations:** ^1^ Key Laboratory for the Physics and Chemistry of Nanodevices and Department of Electronics Peking University Beijing 100871 P. R. China; ^2^ State Key Laboratory for Turbulence and Complex System Department of Mechanics and Engineering Science College of Engineering Peking University Beijing 100871 China; ^3^ Beijing Innovation Center for Engineering Science and Advanced Technology Peking University Beijing 100871 China

**Keywords:** 3D deformable structures, conformable structures, hybrid films, kirigami

## Abstract

By introducing stretchability and/or deformability to planar electronics, devices can conformably attach to 3D curved surfaces with minimal invasiveness, which is of great interest for next‐generation wearables in clinical and biological applications. Here, a feasible route is demonstrated to generate deformable 3D structures as a robust platform to construct electronic systems by utilizing silver nanowires/parylene hybrid films in a way analogous to the art of kirigami. The hybrid films exhibit outstanding electrical conductivity along with decent optical transparency, flexibility, and long‐term stability. These merits enable these films to work as electrodes for electrocardiogram recording with comparable accuracy to a commercial counterpart, and to fabricate a 7‐GHz monopole antenna with good omni‐directionality and a peak gain of 1.35 dBi. More importantly, a general scheme for constructing 3D deformable electronic systems is presented, including unique patterning procedures and rational cut designs inspired by kirigami. As an example, deformable transparent humidity sensors are fabricated to work on elbows and finger joints for sweating monitoring. The strategy demonstrated here for 3D deformable system construction is versatile and holds great promise for future advanced health monitoring at diverse and complex epidermal surfaces.

Diverse health care monitoring, such as heart rate,^1^, ^2^ body temperature,^3^, ^4^ sweat,^5^, ^6^, ^7^ motion,^8^, ^9^ and blood pressure,^10^, ^11^ have been presented plentifully in a soft planar form. However, a more complicated structure is required to achieve an intimate interface with skin for physiological sensing at the convex or concave body areas to enhance signal quality and attenuate noise from the measurement.^12^, ^13^, ^14^, ^15^ Recently, the art of kirigami inspires a way to fashion planar 2D structures into complex 3D structures,^16^, ^17^, ^18^, ^19^, ^20^, ^21^, ^22^ which provides an approach to extend the exceptional implementation capabilities at 2D into 3D. However, wide application of this technology is still hindered by the complex or delicate fabrication procedures.^23^, ^24^, ^25^

In addition to creating an intimate interface, biocompatibility is another indispensable requirement for skin mounted monitoring systems. Parylene stands out as the structural material owing to its chronic implantability (an ISO 10993, United States Pharmacopeia(USP) Class VI material),^26^, ^27^ flexibility, chemical inertness, optical transparency, along with its barrier properties and a conformal deposition process to provide pin‐hole free coating at room temperature.^28^, ^29^, ^30^, ^31^ Meanwhile, physiological signal recording by using silver nanowires (AgNWs) as conducting material has exhibited a huge potential with excellent optical transmittance, low resistance, and good mechanical flexibility.^32^, ^33^, ^34^, ^35^ However, there also exist two remarkable problems to go with AgNWs. One problem is that AgNWs are readily oxidized in air, which results in high surface resistance and thus high skin contact impedance for signal recording.^36^, ^37^, ^38^ The other one is that, when solution deposition or printing method is applied, the adhesion of AgNWs on the supporting substrate is not sufficiently strong to survive multiple attaching/detaching processes on the skin, which leads to a degenerated performance during test.^39^, ^40^ Therefore, a composite consisting of AgNWs and parylene in a specially designed form may provide a strategy to inherit their above‐mentioned advantages while evading the existing problems.^41^

Here, we propose a simple and easy‐to‐implement method to fabricate deformable structures that can be applied to 3D curved surfaces. The schemes involve development of ultrathin AgNWs/parylene hybrid films (with high conductivity, transparency, flexibility, and long‐term stability), pattern design, and geometric transformation from 2D to 3D via laser cutting in a way analogous to kirigami. To verify the excellent properties of the hybrid films, they were applied as electrodes for electrocardiogram (ECG) signal monitoring on the forelimb, showing comparable performance to the commercial ones. Additionally, a 7 GHz monopole antenna was fabricated based on these films that demonstrates quite decent omni‐directionality and a peak gain of 1.35 dBi. Moreover, with a rational pattern design, deformable transparent humidity sensor was processed in a 2D plane and eventually pulled into a 3D adjustable structure to operateon the elbow and finger joint for sweating monitoring, revealing the feasibility of this new proposed approach to achieve high‐performance sensor system working at curvilinear biological surface.

**Figure** **1**a shows a schematic illustration of the fabrication procedures of AgNWs/parylene hybrid films. Two different routes are developed to obtain films as monolithic electrodes (Route 1) or with complex patterned electrodes/interconnectors (Route 2) for different application purposes. AgNWs used here, with an average diameter of 100 nm and a length > 30 µm, were synthesized by a one‐step polyol process at 110 °C^42^ and dispersed in ethanol by ultrasonic waves. To obtain films as monolithic electrodes, AgNWs were first spin‐coated onto a cleaned silicon wafer to form a uniform random distribution of AgNWs networks. Then, a parylene film was deposited on the wafer by chemical vapor deposition at room temperature. Following that, the hybrid film was patterned into designed shape by laser cutting, and peeled off from the silicon wafer as the monolithic electrode. It is suitable for signal recording as a simple conducting pad. To obtain films with complex patterned electrodes/interconnectors, a parylene film was first grown on a cleaned silicon wafer, and shaped into a shadow mask by laser cutting that exposes certain underling areas of silicon wafer. After spin‐coating AgNWs, the patterned parylene layer was tore off, leaving the patterned AgNWs on silicon wafer as electrodes/interconnectors. The subsequent parylene deposition and peeling off allowed these conducting patterns to be transferred into the final parylene film, which is applicable to complex interconnected structures on an insulating substrate for further processing. Figure 1b shows a top‐view scanning electron microscopy (SEM) image of spin‐coated AgNWs dispersed on a silicon wafer with uniform distribution and random orientations. In the hybrid film, due to parylene's conformal, pin‐hole free coating via a gas phase deposition at the molecular level,^43^ AgNWs were mostly buried in parylene, which is revealed by the SEM image shown in Figure 1c and the atomic force microscopy (AFM) image shown in Figure S1 in the Supporting Information. Thus, via both routes, the embedded structure of AgNWs in a 3 µm thick parylene film facilitates long‐term performance stability and good conformal contacts with the skin.

**Figure 1:**
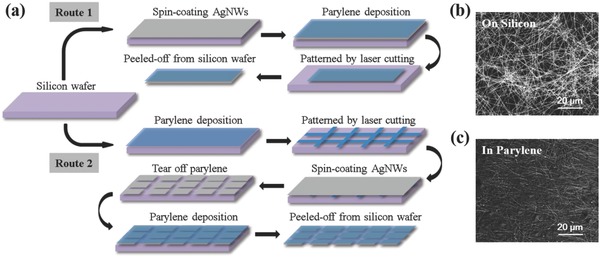
a) Schematic illustration of the fabrication procedures of the AgNWs/parylene hybrid film. SEM image of spin‐coated AgNWs b) dispersed on a silicon wafer and c) buried in parylene film after being transferred.

Properties of the AgNWs/parylene hybrid film were characterized in different aspects, which are summarized in **Figure** **2**. As a spin‐coating process was taken to disperse AgNWs on silicon wafer, the density of the AgNWs on the silicon wafer, and thus the density of the AgNWs in the final hybrid film are related to the concentration of AgNWs solution and the number of spin‐coating times. Four samples, marked as sample 1–4, were prepared by spin‐coating 10 times, 11 times, 12 times, and 13 times of AgNWs solution at a fixed concentration of 1.9 mg mL^−1^, respectively. SEM images in Figure 2a show that the density of AgNWs embedded in parylene film increases gradually with the increased number of spin‐coating times. The duty ratio of AgNWs of samples 1–4 was 14.29%, 16.03%, 17.41%, and 19.97%, respectively. Figure 2b is the light transmittance spectra of the prepared samples in the wavelength range from 350 to 1200 nm with a 3 µm thick parylene film as the reference group. With the increase in the number of spin‐coating times, the transmittance of the hybrid film decreases gradually. The periodic fluctuations observed in the spectra may be caused by optical interference.^44^, ^45^ Figure 2c shows the relationship between sheet resistance and transmittance at 550 nm extracted from Figure 2b of samples 1–4. The sheet resistances reduce from 20.67 to 5.04 Ω/◻ from sample 1 to sample 4, accompanied by a transmittance drop from 77.38% to 69.65%. For a reference, the transmittance of parylene film at 550 nm is 79.84%. It indicates that depending on application purpose, a compromise should be made between conductivity and transparence of the hybrid film.

**Figure 2:**
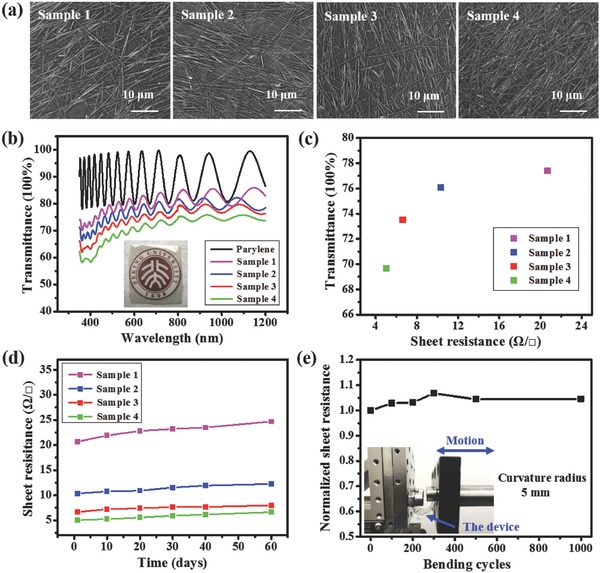
a) SEM images of samples 1–4, showing AgNWs embedded in parylene films with different densities. b) Transmittance spectra of samples 1–4 in the wavelength range from 350 to 1200 nm, using a 3 µm thick parylene film as the reference group. c) The relationship between sheet resistance and transmittance at 550 nm of samples 1–4. d) Changes in sheet resistance of samples 1–4 exposed in air at room temperature. e) Variations in normalized sheet resistance of the hybrid film during bending test. The inset shows the experimental setup.

In addition, long‐term stability and mechanical robustness of these hybrid films were tested. We exposed the hybrid films in air at room temperature and recorded the changes of sheet resistance every 10 d. The test results are shown in Figure 2d. The sheet resistances of samples 1–4 increase by an average of 2.24 Ω/◻ after 60 d, indicating a much better long‐term stability than previous reports.^38^, ^46^, ^47^ This benefits from the embedded structure of AgNWs in parylene, where parylene acts as a barrier layer to protect AgNWs from oxidation in the environment. To test mechanical robustness, the hybrid film was stuck on a polyethylene terephthalate substrate to facilitate operation. Alternate stretching and releasing was introduced into the hybrid film by bending the substrate with a curvature radius of 5 mm via a linear motor at a speed of 20 cm s^−1^ repeatedly. Figure 2e shows the variation of normalized sheet resistance of the hybrid film during cyclic bending test. The sheet resistance increases slightly after the first 300 bending cycles, and it remains stable in the following test. After 1000 bending cycles, the sheet resistance of the hybrid film only increases by 4.53%. The inset of Figure 2e shows the experimental setup for the bending test.

According to the good conductivity and the ultrathin nature for providing conformal contact, the AgNWs/parylene hybrid films were utilized as electrodes, fabricated via Route 1, to monitor ECG signals on the forelimb to verify its capability for physiological monitoring on body surface. Before testing the ECG signal, the skin contact impedance of the electrodes was acquired by placing two electrodes 2 cm apart from each other on the forearm (Figure S2, Supporting Information).^48^ Low impedance values at low frequencies would have a great effect on suppressing electrical interference noise and electronic noise of the electrode itself,^49^, ^50^, ^51^ which is crucial for obtaining artifact‐free electrophysiological recordings.^52^ At 40 Hz, the measured impedance of the AgNWs/parylene hybrid electrodes is 4.7 kΩ when they are attached on the skin for the first time, and increase to 15.6 kΩ after being attached/detached for 20 times, which is still smaller than the impedance of the commercial electrodes of 54 kΩ. This is a satisfying result. **Figure** **3**a shows the working principle for measuring ECG signals. Left and right electrodes were attached to left and right wrists respectively to form a bipolar Limb Lead I derivation.^53^ The electrodes were connected to a low noise voltage preamplifier (SR560, Stanford Research Systems) with a 0.03–30 Hz band‐pass filter in order to eliminate baseline drift (0 Hz) and power line interference (50 Hz).^54^ Figure 3b,c shows ECG signals captured by commercial electrodes and the AgNWs/parylene hybrid electrodes respectively from a healthy male volunteer (age, 22). The ECG signals recorded by the AgNWs/parylene hybrid electrodes showed the similar voltage amplitude and details with the commercial one, indicating a comparable performance. In both sets of charts, characteristic ECG peaks (Q, R, and S) of each wave were clearly recorded.

**Figure 3:**
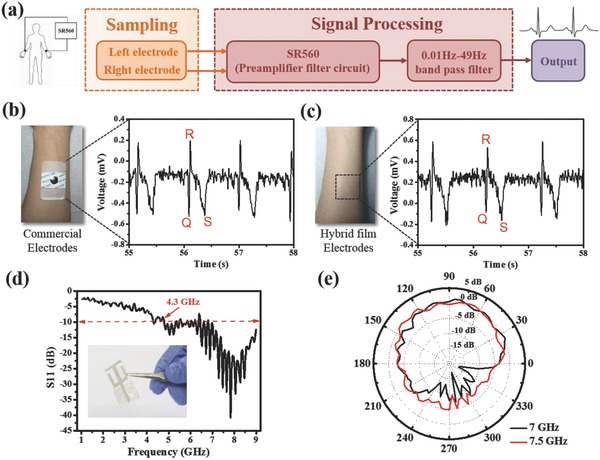
a) The principle for ECG signal recording. ECG signals captured by b) commercial electrodes and c) the AgNWs/parylene hybrid film electrodes from a healthy male volunteer. d) The reflection coefficient response of the monopole antenna over a frequency range from 1 to 9 GHz. The inset shows the photograph of obtained monopole antenna. e) The radiation pattern of monopole antenna in H‐plane at a frequency of 7 and 7.5 GHz.

Wearable sensors for remote diagnostics need to communicate sensory data with information processing terminal, in which the radio frequency antenna is a key component. The higher the frequency is achieved, the smaller the footprint of the antenna and thus a better integration capability could be. In our case, the excellent electrical conductivity (similar to metal materials) of AgNWs and the low dielectric constant and dissipation factor of parylene confer initial advantages for fabricating super high radio frequency antennas. A 7 GHz monopole antenna was successfully realized via Route 2, consisting of a modified dual G‐shaped radiating strip (based on AgNWs), a ground plane (based on AgNWs), and a uniform layer of dielectric substrate (parylene) sandwiched between them. Figure 3d shows the reflection coefficient response of the monopole antenna over a frequency range from 1 to 9 GHz. The inset shows a photograph of the obtained monopole antenna with a size of 37 mm × 18 mm and a total thickness of 3 µm. The operating frequency range of the antenna (S11 < −10 dB) is very wide, from 4.3 to 9 GHz, including the main operating frequency bands of WLAN (5 GHz) and RFID (4.3–5.8 GHz). The radiation pattern for the monopole antenna was monitored in a far‐field microwave anechoic chamber at a frequency of 5, 5.5, 6, 6.5, 7, 7.5, and 8 GHz, respectively (Figure S3, Supporting Information). Among them, the radiation pattern in the H‐plane at frequency of 7 and 7.5 GHz are shown in Figure 3e. The antenna exhibits excellent omnidirectional radiation characteristics with a peak gain of 1.35 dBi at 7 GHz. Compared with previous works,^55^, ^56^, ^57^ the antenna obtained here achieves a higher frequency with minimized footprint, which has the potential in applications of wireless communications for sensing systems.

To introduce deformability to planar electronic system for providing a conformal contact with curved biological surfaces, we developed a general scheme to extend a system in a 2D plane into a 3D adjustable form by unique patterning procedures and rational cut designs inspired by kirigami, which took the advantages both from the superior fabrication capabilities existing for planar system and the compliant mechanism serving by the deformability. **Figure** **4**a–d shows two examples of patterning the hybrid film in a 2D plane and then being pulled into an out‐of‐plane shape‐adjustable cone structure by tweezers manually in schematic ways and with photos recorded during the pulling processes. No significant mechanical damages were observed during the shape transformation. To provide guidance or justification for the design of the kirigami pattern, a theoretical analysis was carried out. In real applications, the hybrid film's deformed contour (determined by the human body) will have an average radius of curvature in the order of centimeters, much larger than the device thickness (3 µm). Therefore, the bending energy is negligible. The device deforming with the human body can be simplified as the 2D problem as illustrated in Figure S4a (Supporting Information)—a thin film attached to the elastic substrate under in‐plane tension. Through the shear‐lag analysis in Note 1 of the Supporting Information, we show that the adhesion between the device and human body is size‐dependent. When the width of the film patterns *L*_0_ reduces, the maximum strain at which the film detaches from the substrate increases (see Figure S4b in the Supporting Information). This justifies the advantage of patterning the device—it can effectively enhance the adhesion between the device and the human body. Furthermore, it provides some guidance to pick up a proper feature size of the kirigame pattern depending on the shape of the 3D surface the device tries to conform to, which is verified in principle by tensile experiments on two parylene‐PDMS specimens shown in Figure S4c in the Supporting Information.

**Figure 4:**
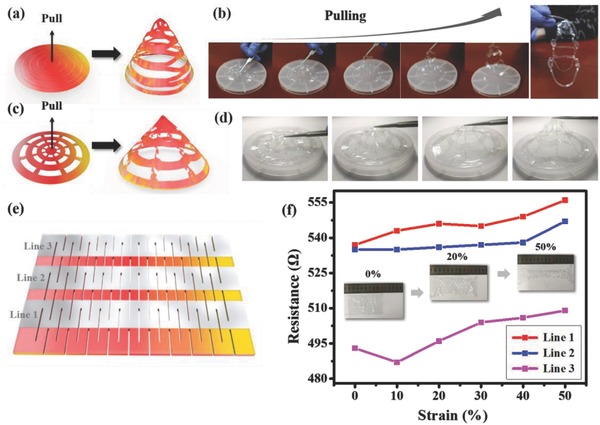
a–d) Schematic images and photos recorded during pulling processes showing two examples of patterning hybrid film in a 2D plane and then being pulled into a 3D structure. e) A simple kirigami pattern consisting of straight lines in a centered rectangular arrangement. f) Variations in resistance of three AgNWs interconnections under different strain conditions. The inset shows the shape change of the hybrid film at 0%, 20%, and 50% strain, respectively.

In order to explore the conductivity change of the hybrid film during deformation process, a kirigami pattern consisting of straight lines in a centered rectangular arrangement provide a simple example. Three interlaced straight lines of AgNWs were patterned via Route 2, as shown in Figure 4e. Figure 4f shows the change in resistance of three AgNWs interconnectors under different strain conditions. The inset shows the shape change of the hybrid film at a strain of 0%, 20%, and 50%, respectively. The original size of the entire device before stretching is 90 mm × 50 mm. The initial resistances of lines 1–3 are 537, 535, and 493 Ω, respectively. With the increase of strain, the resistance of line 1–3 changes slightly. When the strain reaches 50%, the resistance of lines 1–3 reached 556, 547, and 509 Ω, respectively, where an average change of 3% is obtained, showing a good mechanical stability of the 3D adjustable hybrid film.

Furthermore, we utilized the 3D adjustable cone structure to build a deformable humidity sensor and achieved sweat monitoring on the curved body surface successfully. **Figure** **5**a shows a schematic illustration of the fabrication procedure of the humidity sensors. Patterned AgNWs electrodes were prepared on silicon wafer by using parylene film as mask, which is similar to that of the previous antenna preparation process (Route 2). After a 3 µm thick parylene film was deposited on the patterned AgNWs electrodes, the AgNWs/parylene hybrid film was cut with a designed pattern by a laser cutting system. After the patterned AgNWs/parylene hybrid film was peeled off from the wafer, sodium polystyrenesulfonate (NaPss) and carboxymethylcellulose (CMC) solution with a mass fractions of 0.02% was dip‐coated on the AgNWs electrodes.^58^ Figure 5b shows that the sensor is attached to the curved skin of finger joint and elbow, realizing conformal contact to these surfaces. The humidity sensor was calibrated first prior to sweating monitoring, and the calibration procedure is depicted in Figure 5c. Conductivity values of the sensor in different relative humidity (RH) environments were obtained with saturated aqueous solutions of different salts respectively, and subsequently fitted with these experimental values to obtain the conductance–humidity fitted curve, as shown in Figure 5d. Then, sweating condition of the human body was monitored by the attached humidity sensor on the elbow. Initially, when the skin is dry, the conductance of the humidity sensor is measured to be 4.38 × 10^−10^ S, which corresponds to a RH of 26.0%. After exercise, RH on the body surface of the wearer increases to 57.3%. With accumulative time of exercise and sweating, the humidity sensor monitored RH increases further to 66.1%. As a proof of concept, the 3D adjustable structure has shown a good example of providing a general platform for physiological signal monitoring on 3D curved body surfaces.

**Figure 5:**
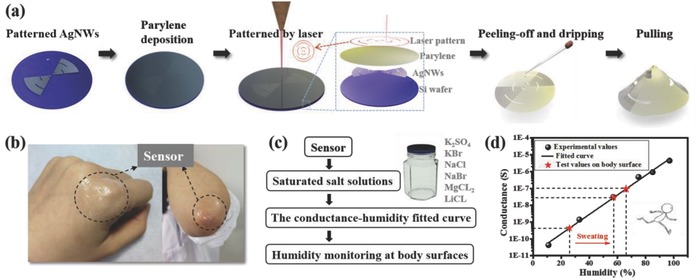
a) Schematic illustration of the fabrication procedure of a 3D humidity sensors. b) Photos showing sensors attached to the curved skin of the finger joint and elbow. c) The calibration procedure of the humidity sensor. d) The conductance–humidity fitted curve of the humidity sensor and the sweating conditions monitored on the elbow.

By combining material design and fabrication strategy, we have developed a scheme to transform planar electronic systems into mechanically tunable 3D form with the capability to work on curved biological surface. The incorporation of conductive nanomaterials and ultrathin barrier film with kirigami art provides a synergy in material properties and system design flexibility. More complex 3D electronic systems that take fully advantage of exceptional planar fabrication capability via this scheme would be a possible solution for emerging wearables in clinical and biological applications.

## Experimental Section

*Preparation of AgNWs/Parylene Hybrid Film*: AgNWs were synthesized by a one‐pot reaction.^42^ Briefly, polyvinylpyrrolidone (0.2 g) was added to ethylene glycol (EG, 25 mL). Then, AgNO_3_ (0.25 g) and FeCl_3_ salt solution (3.5 g, 600 × 10^−3^ m in EG) were added. Complete dissolution was required to obtain a transparent and uniform solution by using magnetic stirring at room temperature. The mixture was then reacted at 110 °C for 12 h. Afterward, the precipitate was washed with acetone and ethanol with centrifugation of 4000 rpm for 5 min. The obtained AgNWs were dispersed in ethanol and were spin‐coated onto a cleaned silicon wafer at 200 rpm. Subsequently, a parylene layer (3 µm) was deposited on the wafer by chemical vapor deposition (SCS Labcoter 2, Specialty Coating Systems. Inc.) at room temperature. Following that, the hybrid film was patterned into designed shape by a laser cutting system (PLS6MW, Universal Laser Systems). Last, the hybrid film was peeled off from the silicon wafer.

*Characterization of the Hybrid Film*: The morphological study of the AgNWs networks was investigated by field‐emission SEM (Quanta 600F, FEI). The transmittance spectra were measured with an UV–visible spectrometer (Cary 5000, Varian) in the spectral range from 300 to 1200 nm. The sheet resistances of the films were measured by a multifunction digital four‐probe tester (ST‐2258C, JG Electronic). The bending test was performed by bending the sample via a constant‐speed linear motor (E1100, LinMot) repeatedly.

*ECG Monitoring*: The commercial ECG electrodes were purchased from Shanghai Jun Kang Medical Equipment Co. Ltd. ECG signals were measured by a low‐noise preamplifier (MODEL SR560, Stanford Research Systems). All experiments with human subjects are performed in compliance with the relevant laws and institutional guidelines. The institutional committee and the human subject have approved the experiments.

*Test of AgNWs/Parylene‐Based Monopole Antennas*: After the AgNWs/parylene‐based monopole antenna was peeled off from the substrate, the antenna was connected to a coaxial cable via a SMA connector with the aid of silver paste (Ted Pella, Inc.). The reflection coefficients at frequencies from 1 to 9 GHz were collected using a vector network analyzer (E8361C, Agilent). The radiation patterns were measured in a far‐field microwave anechoic chamber, which contained a network analyzer (AE5071C, Agilent).

*Fabrication and Calibration of the Wearable Humidity Sensor with a 3D Structure*: To prepare the humidity sensor, NaPss and sodium CMC were utilized as humidity‐sensitive materials.^58^ First, NaPss and CMC (1:1) were dissolved in DI water with a mass fraction of 0.02% to obtain the polymer solution. Then the solution was dip‐coated on the AgNWs electrodes, and baked at 80 °C for 30 min to cure the NaPss and CMC. Saturated aqueous solutions of K_2_SO_4_, KBr, NaCl, NaBr, MgCl_2_, and LiCl provided RHs of 97%, 85%, 75%, 57%, 33%, and 11%, respectively, for calibration of the humidity sensor.

## Conflict of Interest

The authors declare no conflict of interest.

## Supporting information

Supplementary
