# Supplementary material for: Kirigami‐Inspired Deformable 3D Structures Conformable to Curved Biological Surface
Source: Adv Sci (Weinh). 2018 Oct 16;5(12):1801070. doi: 10.1002/advs.201801070 (PMC6299731; doi:10.1002/advs.201801070)
Supplement: Supplementary file 1 — Supplementary [file ADVS-5-1801070-s001.pdf]

Copyright WILEY-VCH Verlag GmbH & Co. KGaA, 69469 Weinheim, Germany, 2018.

ADVANCED SCIENCE
Open Access

Supporting Information

for *Adv. Sci.,* DOI: 10.1002/advs.201801070

Kirigami-Inspired Deformable 3D Structures Conformable to Curved Biological Surface

*Chao Yang, Heng Zhang, Youdi Liu, Zhongliang Yu, Xiaoding Wei, and Youfan Hu**

Copyright WILEY-VCH Verlag GmbH & Co. KGaA, 69469 Weinheim, Germany, 2016.

Supporting Information

Kirigami-Inspired Deformable 3D Structures Conformable to Curved Biological Surface

*Chao Yang, Heng Zhang, Youdi Liu, Zhongliang Yu, Xiaoding Wei, Youfan Hu**

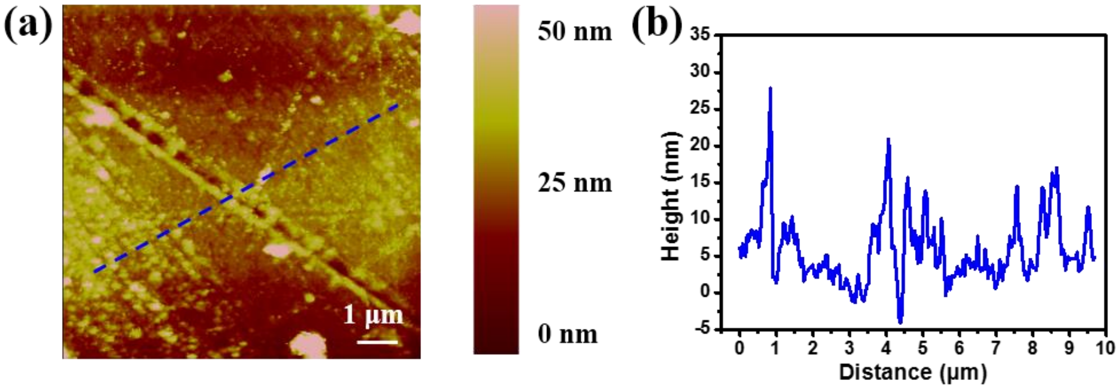

Figure S1. (a) AFM image of the surface of AgNWs/parylene hybrid film. (b) The height profile measured along the dotted line in (a). The measured maximum height is about 27 nm with an average roughness of 6.66 nm, which is far smaller than the diameter of AgNWs of 100 nm.

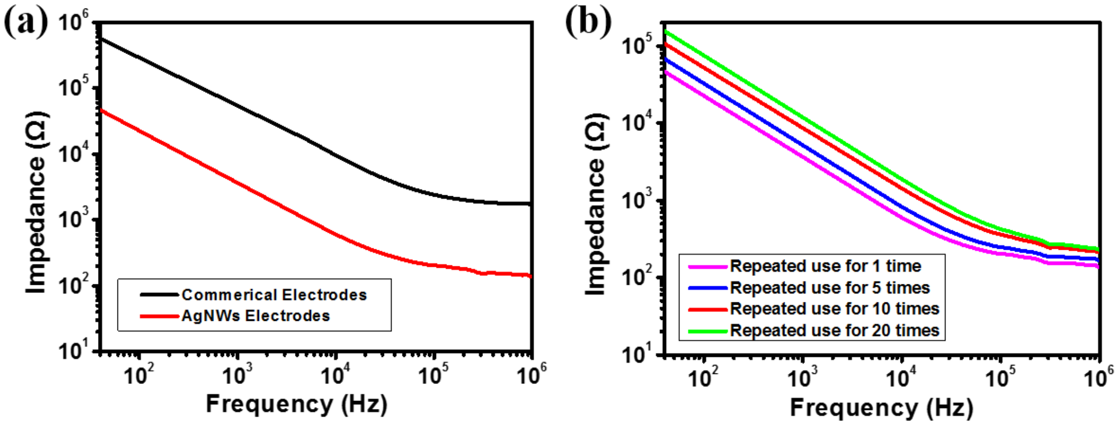

Figure S2. (a) Impedance of the skin/electrode interface. AgNWs/parylene hybrid film electrodes (red) and commercial electrodes used for reference (black) were attached onto the skin of the forearm. (b) Impedance of the skin/electrode interface after the AgNWs/parylene hybrid film electrodes were repeatedly used for 1 time, 5 times, 10 times and 20 times. The impedance spectra measured from 40 Hz to 1 MHz were acquired using a precision impedance analyzer (4294A, Agilent) with two electrodes placed 2 cm apart from each other. At 40 Hz, the impedance of the AgNWs/parylene hybrid film electrodes was 4.7 kΩ, which is smaller than the impedance of commercial electrodes of 54 kΩ. Both values of impedance are sufficiently low to measure EMG signals without significant noise. After the AgNWs/parylene hybrid film electrodes were repeatedly used for 1 times, 5 times, 10 times and 20 times, the impedance was slightly increased. Among them, after repeated use for 20 times, the impedance increases to 15.6 kΩ at 40 Hz.

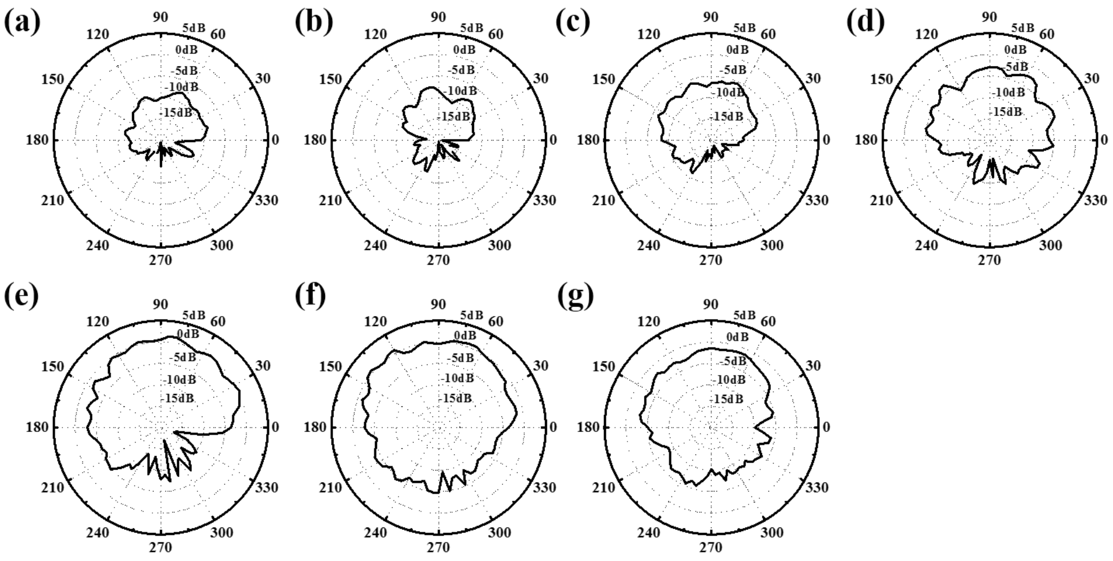

Figure S3. The radiation pattern of the monopole antenna in the H-plane at frequency of (a) 5 GHz, (b) 5.5 GHz, (c) 6 GHz, (d) 6.5 GHz, (e) 7 GHz, (f) 7.5 GHz and (g) 8 GHz. As the frequency increases, the radiation characteristics of the monopole antenna become better. The peak gain of the monopole antenna increases from -7.85 dBi at 5 GHz to 1.35 dBi at 7 GHz. With the frequency continues to increase, the antenna exhibits excellent omnidirectional radiation characteristics with a peak gain of 0.88 dBi at 7.5 GHz.

Note 1. Size-dependent adhesion performance

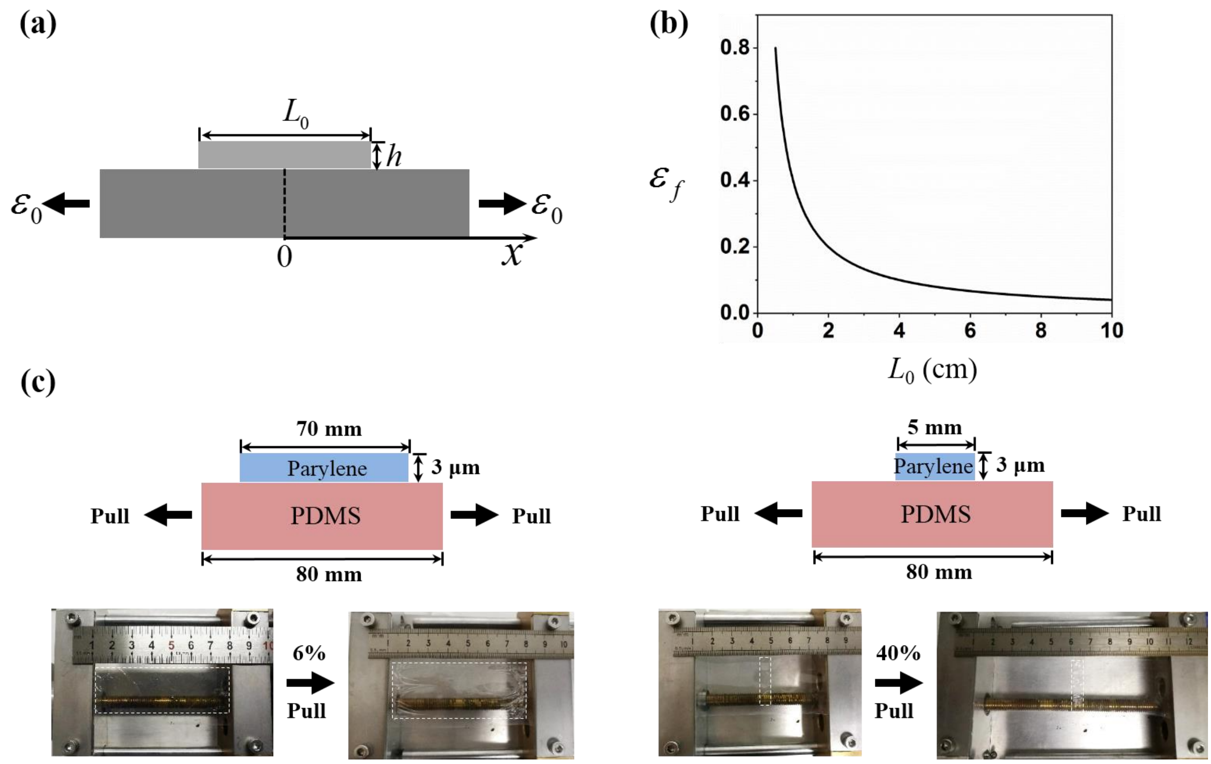

Figure S4. Size-dependent adhesion between film and substrate. (a) A simplified model for the hybrid film and substrate under uniaxial tension. (b) Plot shows how the strain corresponding to the hybrid film debonding from human skin varies with the length of hybrid film. (c) Tensile experiments on film-substrate specimens with 70 mm film width and 5 mm film width, respectively, to compare their debonding strains. White rectangles highlight the parylene film locations in each snapshot.

Considering the device thickness is much less than the radius of curvature during its deformation with the human body, we can neglect the energy contribution from film bending and simplify the problem into the in-plane mechanical model shown in Figure a. The device is modeled as a thin film (with width L_0 and thickness h) attached on the substrate (*i.e.*, the human skin). The film and substrate are assumed to behave linear elastically. The substrate is subjected to a tensile strain ε_0. The film-structure interface will transfer the tension to the thin film through the shear stress. Based on the classical shear-lag model[1,2], the infinitesimal analysis on the hybrid film gives the force equilibrium as

$$\tau(x) = -h\frac{d\sigma(x)}{dx}, \tag{1}$$

where $\sigma(x)$ is the normal stress in the hybrid film, and $\tau(x)$ is the shear stress along the interface. Since $\sigma(x) = E\,\varepsilon(x)$, where E and $\varepsilon(x)$ are the elastic modulus and tensile strain of the thin film, Eq. (1) can be rewritten as

$$\frac{d\tau(x)}{dx} = -Eh\frac{d^2\varepsilon(x)}{dx^2} \tag{2}$$

Assuming a linear interface (*i.e.*, $\tau(x) = K\delta$, where K and δ are the stiffness and relative displacement between the thin film and substrate), Eq. (2) can be expressed as

$$\frac{d^2\varepsilon(x)}{dx} - \lambda^2\varepsilon(x) + \lambda^2\varepsilon_0 = 0. \tag{3}$$

where $\lambda = \sqrt{K/(Eh)}$. The boundary conditions read:

$$\begin{cases} \varepsilon(x)\big|_{x=L_0/2} = 0 \\ \left.\dfrac{d\varepsilon(x)}{dx}\right|_{x=0} = 0 \end{cases}. \tag{4}$$

The solution to Eqs. (3) and (4) is

$$\varepsilon(x) = \varepsilon_0\left[1 - \frac{\cosh(\lambda x)}{\cosh(\lambda L_0/2)}\right]. \tag{5}$$

Then, integrating Eq. (2) with the strain distribution given by Eq. (5) yields the interfacial shear stress distribution:

$$\tau(x) = \varepsilon_0\lambda Eh\frac{\sinh(\lambda x)}{\cosh(\lambda L_0/2)}. \tag{6}$$

Thus, the relative displacement at the interface reads

$$\delta(x) = \frac{\varepsilon_0\lambda Eh\sinh(\lambda x)}{K\cosh(\lambda L_0/2)}. \tag{7}$$

On the other hand, the strain energy release rate at the interface G_c is $G_c(x) = \frac{1}{2}\tau(x)\delta$.

Combining Eqs. (6) and (7), the energy release rate at the two ends of the film is

$$G_{c,end} \equiv G_c\big|_{x=L_0/2} = \frac{\varepsilon_0^2\lambda^2E^2h^2}{2K}\tanh^2\left(\lambda L_0/2\right). \quad (8)$$

When the energy release rate at the film ends reaches the film-substrate adhesion energy (*i.e.*, $G_{c,end} = G_{IIc}$), the film starts to detach from the substrate. The tensile strain applied on the substrate is identified as the debonding strain ε_f:

$$\varepsilon_f = \frac{\sqrt{2G_{IIc}K}\coth\left(\lambda L_0/2\right)}{\lambda Eh}. \quad (9)$$

Clearly, the above equation suggests a strong size effect in the debonding strain. The device thickness in this study is h=3 μm. The (AgNWs)/parylene hybrid film has an elastic modulus of approximately 10 *GPa*. Assuming the adhesion energy and stiffness of the device-skin interface as $G_{IIc} = 1J/m^2$ and K=0.5*MPa/m,* respectively, the debonding strain is plotted in Figure b as a function of the segment width L_0. For a continuous device without patterning, L_0~70 mm. After patterning, the segment width is approximately 5 mm ~ 12 mm. Our model prediction suggests that the debonding strain increases from less than 10% for the continuous device to approximately 40% after patterning. To verify our model prediction, we carried out tensile experiments on two parylene-PDMS specimens as shown in Figure S4c. The only difference between two specimens is the parylene film width – the wide specimen has L_0 = 70 mm, and the narrow specimen has L_0 = 5 mm. The experimental results show that the parylene film detached from the PDMS substrate at a tensile strain of only 6%. In comparison, the narrow specimen did not show plausible detachment from the substrate even at 40% tensile strain. The experimental observation agrees well with the trend of adhesion performance predicted by our analysis. Thus, the model and experiments confirm that patterning the device significantly helps the adhesion performance. Recently, a similar phenomenon was reported on mussel adhesion – research found that discrete interfaces could greatly enhance the adhesion[3].

References

[1] H. Cox, *British journal of applied physics* **1952**, *3*, 72.
[2] T. Jiang, R. Huang, Y. Zhu, *Advanced Functional Materials* **2014**, *24*, 396.
[3] A. Ghareeb, A. Elbanna, *Journal of Applied Mechanics* **2018**, *85*, 121003.
